# Long-term exposure to air pollution and hospital admissions for ischemic stroke. A register-based case-control study using modelled NO_x _as exposure proxy

**DOI:** 10.1186/1471-2458-9-301

**Published:** 2009-08-19

**Authors:** Anna Oudin, Emilie Stroh, Ulf Strömberg, Kristina Jakobsson, Jonas Björk

**Affiliations:** 1 Department of Occupational and Environmental Medicine, Lund University Hospital, Lund, Sweden; 2 Competence Centre for Clinical Research, Lund University Hospital, Lund, Sweden

## Abstract

**Background:**

Long-term exposure to air pollution is a hypothesized risk factor for ischemic stroke. In a large case-control study with a complete study base, we investigated whether hospital admissions for ischemic stroke were associated with residential concentrations of outdoor NO_x_, as a proxy for exposure to air pollution, in the region of Scania, Southern Sweden.

**Methods:**

We used a two-phase case-control study design, including as first-phase controls all individuals born between 1923 and 1965 and residing in Scania in 2002 (N = 556 912). We defined first-phase cases as first-time ischemic stroke patients residing in Scania and registered in the Swedish stroke register between 2001 and 2005 (N = 4 904) and second-phase cases as cases for whom we had information on smoking status, diabetes, and medication for hypertension (N = 4 375). For the controls, information on these covariables was collected from a public health survey, resulting in 4 716 second-phase controls. With a geographical information system and an emission database, individual residential outdoor annual mean NO_x _concentration was modelled. The data were analyzed with logistic regression.

**Results:**

We found no evident association between NO_x _and ischemic stroke. For example, the odds ratio for ischemic stroke associated with the NO_x _category 20–30 μg/m^3 ^compared to the reference category of <10 μg/m^3 ^was 0.95 (95% CI 0.86–1.06).

**Conclusion:**

In this study area, with generally low levels of air pollution, using a complete study base, high-quality ascertainment of cases, and individually modelled exposure, we did not observe any clear association between NO_x _and ischemic stroke hospital admissions.

## Background

It is well established that air pollution can influence health.[1,2] Evidence has been presented that high levels of air pollution have trigger (short-term) effects on ischemic stroke mortality, [3] and on hospital admissions for ischemic stroke, [4,5] although other studies have been negative.[6] Rather low levels of air pollution have been claimed to have short-term effects on the risk of ischemic stroke.[7] One study found moderate short-term association between low levels of air pollution and hospital admissions for all cerebrovascular diseases, but no association was found for cerebral ischemic diseases.[8]

There have been considerably fewer studies on long-term effects of air pollution on ischemic stroke risk than studies on short-term effects. In England, Maheswaran and colleagues found that living near main roads (as an indicator for long-term exposure to air pollution) increased stroke mortality,[9] as did long-term exposure to NO_x_, PM_10 _and CO.[10] In an American study, PM_2.5 _was observed to be a risk factor for cerebrovascular events in women.[11] A Norwegian study in men found long-term associations between a 10 μg/m^3 ^increase in NO_x _and all-cause mortality, but for cerebrovascular mortality, an association was not evident.[12] The studies on long-term exposure to air pollution and stroke have not differentiated ischemic stroke from other cerebrovascular events.

The biological mechanism between air pollution and risk of stroke is not yet clearly established, and the mechanism might differ between short-term and long-term effects. However, evidence has been presented that high levels of air pollution can alter the coagulation of the blood,[13] that long-term exposure to air pollution increases the risk of atherosclerosis[14] and that diesel exhaust exposure increases thrombus formation in man.[15] Moreover, long-term exposure to particulate air pollution has recently been observed to increase the risk of deep vein thrombosis.[16]

**Figure 1:**
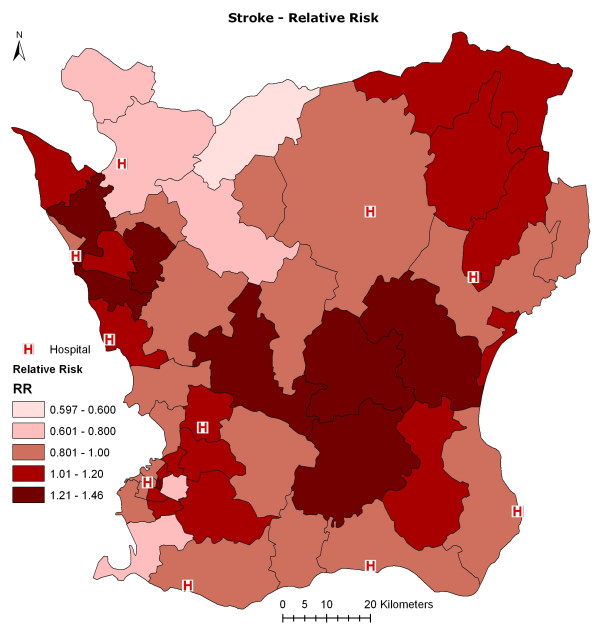
**Relative risk of ischemic stroke in Scania, modelled with a Rapid Inquiry Facility (RIF) Software, geographically distributed over 52 strata**.

The variation in relative risk of ischemic stroke hospital admissions in Scania is high between different areas (Figure 1). Our main objective was to determine whether air pollution might be associated with this variation in ischemic stroke incidence: adjusted for smoking and other important potential confounders. We used information on registered hospital-treated ischemic stroke cases in the region during 2001 to 2005 in a two-phase case-control study design, where the entire population born between 1923 and 1965 and residing in Scania in 2002 was included as controls in the first phase. Modelled annual mean outdoor concentrations of NO_x _(a mixture of nitrogen oxide and nitrogen dioxide) at the study subjects' residential addresses were used as a marker of exposure to air pollution. The low levels of air pollution were of particular interest. The NO_x _annual mean of 12.5 μg/m^3 ^in Scania in this study are well below the WHO guidelines of an NO_2 _annual mean of 40 μg/m^3^.[17]

## Methods

### Study area

#### Scania

The study area, Scania, is the southernmost region in Sweden. It covers 11 350 km^2^, which is approximately 2% of the Swedish land area.[18] In December 2008, Scania had around 1.2 million habitants, approximately 13% of the Swedish population.[19] Levels of pollutants in Scania are generally higher than in Sweden overall, but rather low from an international point of view.[17]

### Cases

The study was based on a series of 14 770 hospital admitted cases of stroke that occurred between January 1, 2001 and December 31, 2005 in the region of Scania, which we obtained data on from Riks-stroke, the national quality register for acute stroke (national stroke register). This register has been described in detail elsewhere[20,21] and has an estimated coverage of hospital admitted ischemic stroke cases of around 90%. However, the register does not include stroke patients who die out of hospital before the stroke is registered. A study in one of the hospital admission areas in Scania suggests that 1% of the stroke patients had a fatal stroke before a medical examination and thus were not included in the register.[22] Moreover, that study showed that a subgroup of male patients with mild strokes were to a larger extent sent home without being registered (8% of the total patients). To obtain as high quality a case definition as possible, we chose not to incorporate information from the cause-of-death register. The outcome in this study is therefore ischemic stroke hospital admissions.

**Figure 2:**
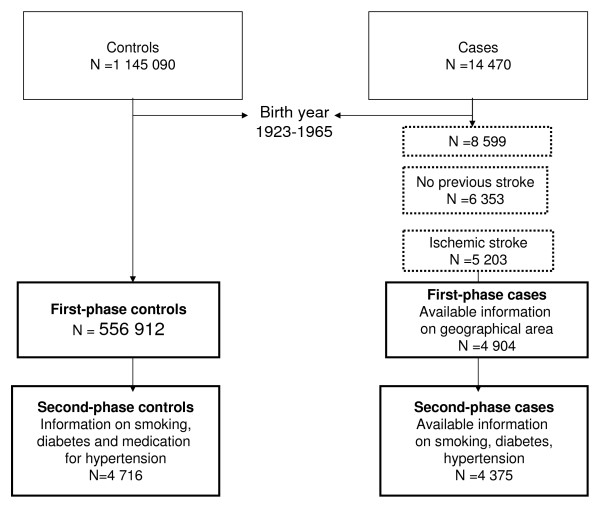
**Flow-chart for cases and controls**.

First-time episodes of ischemic stroke in persons born between 1923 and 1965 were included, resulting in 4 904 first-phase cases (Table 1 and Figure 2). The median age at diagnosis was 72 years.

**Table 1:** First-phase descriptive data for the variables birth year category, sex, birth country and marital status.

		Cases N (%)	Controls N(%)	OR^b^	95% CI	Controls, stratified by modelled residential outdoor NO_x_-level^a^	
		
						≤20 μg/m^3^ NO_X_ N (%)	>20 μg/m^3^ NO_X_ N (%)
						
Birth year	1923–1925	970 (20)	24 647 (4)	56	43–73	17 773 (4)	6 874 (5)
	1926–1930	1 262 (26)	45 059 (8)	40	30–52	33 078(8)	11 981 (9)
	1931–1935	876 (18)	47 814 (9)	26	20–34	35 590(8)	12 224(9)
	1936–1940	682 (14)	56 671 (10)	17	13–23	43 071(10)	13 600 (10)
	1941–1945	523 (11)	74 743 (13)	9.9	7.5–13	58 002(14)	16 741 (12)
	1946–1950	286 (6)	80 349 (14)	5.0	3.8–6.7	61 900(15)	18 449 (13)
	1951–1955	169 (3)	73 802(13)	3.2	2.4–4.4	55 545(13)	18 257 (13)
	1956–1960	79 (2)	73 277(13)	1.5	1.1–2.1	54 694(13)	18 583 (14)
	1961–1965	57 (1)	80 550(14)	1		59 699(14)	20 851 (15)
							
Sex	Male	2 778 (57)	273 489(49)	1.3	1.3–1.4	207 222(49)	66 267 (48)
	Female	2 126 (43)	283 423(51)	1		212 130(51)	71 293 (52)
							
Birth country	Nordic	213 (4)	20 238(4)	0.82	0.75–0.90	14637(3)	5601 (4)
	Other	501 (10)	68 325(12)	1.2	1.0–1.4	34 832 (8)	33 493 (24)
	Sweden	4 190 (85)	468 349(84)	1		369 893(88)	98 466 (72)
							
Marital status	Divorced	895 (18)	95 811 (17)	1.2	1.1–1.3	64 031 (15)	31 780 (23)
	Widowed	985 (20)	36 155(6)	3.6	3.3–3.9	26 163 (6)	9 992 (7)
	Not married	495 (10)	92 016 (17)	0.71	0.64–0.78	64 211(15)	27 805 (20)
	Married	2 529 (52)	332 930(60)	1		26 4947(63)	67 983 (49)

^a^The residential outdoor annual means of NO_X_, modelled with a GIS and an emission database. The year for the annual mean is selected by distributing the controls over time with the same distribution as the date of diagnosis for the cases.

^b ^Crude odds ratio for ischemic stroke.

The proportion of computer tomographies among our stroke cases was 99% for ischemic stroke cases and 97% in total, which indicates an accurate classification of types of stroke.

Out of the 4904 first-phase cases, 1552 (32%) were registered as deceased the 29^th ^of June 2009. Out of the 1552 deceased, 152 (10% of the deceased, 3% of the total number of cases) died within 10 days of the day the stroke occurred, 244 (16%, 5%) died within 30 days after the stroke and 438 (28%, 9%) died within 6 months after the stroke.

The national stroke register provided information on smoking status, diabetes, and medication for hypertension for 4 375 of the 4 904 ischemic stroke cases (Table 2, Figure 2). Statistics Sweden, which is a central government authority for registration and provision of official statistics, provided information on the marital status and country of birth of all cases.

**Table 2:** Second-phase descriptive data for the variables diabetes, smoking, medication for hypertension.

		Cases	Controls	OR^b^	95% CI	Controls, stratified by modelled residential outdoor NO_x_-level^a^	
						
		N (%)	N(%)			≤20 μg/m^3 ^NO_X_ N (%)	>20 μg/m^3^ NO_X_ N (%)
		
Smoking	Yes	1139 (26)	629 (13)	2.8	2.5–3.2	457 (12)	172 (17)
	No	3236 (74)	4087 (87)	1		3250 (88)	837 (83)
							
Diabetes	Yes	934 (21)	501 (11)	2.1	1.8–2.4	388 (10)	113 (11)
	No	3441 (79)	4215 (89)	1		3319 (90)	896 (89)
							
Medication for hypertension	Yes	1974 (45)	1482(31)	1.7	1.5–1.8	1161 (31)	321 (32)
	No	2401(55)	3234 (31)	1		2546 (69)	688 (68)
							
Total		4375	4716				

^a^The residential outdoor annual means of NO_X_, modelled with a GIS and an emission database. The year for the annual mean is selected by distributing the controls over time with the same distribution as the date of diagnosis for the cases

^b ^Crude odds ratio for ischemic stroke.

### First-phase Controls

Information on year of birth, sex, country of birth, marital status, and location of residence was available from Statistics Sweden. The information was dated December 31, 2002. Individuals born between 1923 and 1965 who were residing in Scania were included, which resulted in 556 912 controls (Table 1, Figure 2).

We stratified the controls by exposure (NO_x _above or below 20 μg/m^3^) to see if potential risk factors were associated with exposure level. This stratification suggested that country of birth and marital status might confound unadjusted estimates of exposure effect (Table 1).

### Second-phase controls

Information on smoking status, diabetes, and medication for hypertension was obtained for controls that had participated in a large public health survey distributed as a mailed questionnaire in 2004. The target population of the survey was individuals between 18 and 80 years of age who were residing in Scania on June 30, 2004. The recruiting was sampled on geographical area. In total, 27 963 people responded to the questionnaire, which corresponds to an overall participation rate of 59%. There was selective participation in the public health survey with respect to income, age, education, sex, marital status and country of birth. The most pronounced difference in participation was between people born outside Europe (33.9%) and people born in Sweden (59.5%).[23] A group-level negative correlation between degree of participation and air pollution was present, but data did not allow investigating if it was also present on the individual level.

We matched each ischemic stroke case to two controls (or if two were not available, to one control) from the public health survey. We matched on birth year category, sex, and on the geographical areas used in the public health survey, resulting in a total of 5 333 controls (Figure 2). Of these controls, 4 716 had complete information on smoking status, diabetes, and medication for hypertension (Table 2, Figure 2).

### Exposure assessment

Assessment of individual levels of exposure to air pollution is challenging,[24] but modelling of air pollution using geographic information systems (GIS) together with information on emissions, meteorology, and traffic intensity enables one to model levels of air pollution with high resolution, in terms of both geography and time.

As a marker of exposure to air pollution, we chose NO_x_, which is considered to be a good proxy for air pollution in general, especially for substances generated by traffic.[2] We used a GIS linked to an emissions database with approximately 24 000 sources of emissions to model individual residential levels of NO_x _outdoors.

The modelled concentrations were validated against measurements from 23 monitoring sites in Scania, yielding an overall Pearson correlation coefficient with the modelled values of r = 0.69.[18] The correlation between the measured concentrations and the error (difference between measured and modelled concentration) was r = -0,016 and between the modelled concentrations and the error r = 0.55, suggesting a classical measurement error structure.[25] The standard deviation of the measured values was 7.7 μg/m^3 ^and of the modelled values 8.3 μg/m^3^.

**Figure 3:**
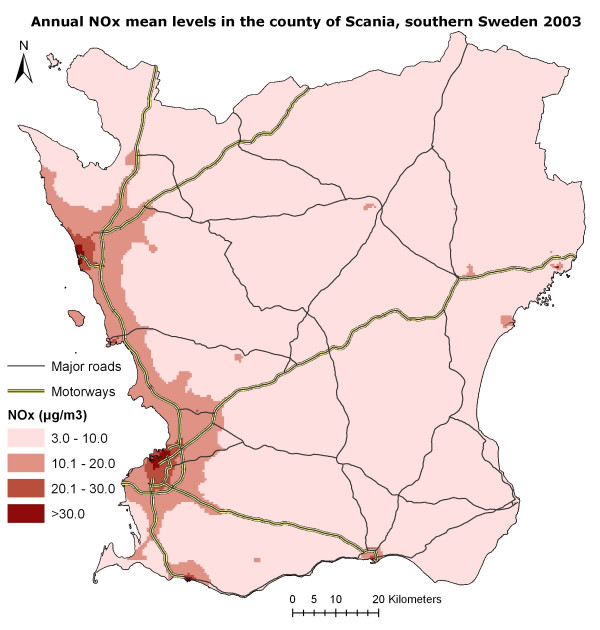
**The distribution of the modelled NO_x _concentrations in Scania**. The mean NO_x _concentration for the cases was 12.9 μg/m^3^, with a standard deviation of 7.8, and for the controls 12.5 μg/m^3^, with a standard deviation of 8.0.

The modelled annual means of NO_x _in Scania are presented in Figure 3. We modelled NO_x _on an hourly basis in a grid with a spatial resolution of 500 × 500 m. [Susanna Gustafsson, A geographical and temporal high resolution database for dispersion modelling of environmental NO_x _in Southern Sweden, submitted] A previous study indicated that a resolution of 200–400 m is suitable in urban areas, but the larger size of our study made 500 × 500 m a feasible choice in spatial resolution.[26] Also, in an ongoing study, measured data is compared with modelled data using different spatial resolutions. Preliminary validation results from that study indicate that a resolution of 500 × 500 m and a resolution of 250 × 250 m yield rather similar results [Emilie Stroh, Personal communication]

For the cases, the individual geocoded residential addresses on December 31 of the year before diagnosis of ischemic stroke were linked to exposure data, and exposure was assessed as the annual mean (at that address) before the date the stroke occurred. The first-phase controls were linked to the address they had on December 31, 2002. The second-phase controls were linked to the address they had in the public health survey of 2004. The controls were randomly distributed over the days of the study period (2001–2005), with the same distribution as the ischemic stroke cases, and their annual mean exposure was assessed as the annual mean before that randomly assigned date.

Although our aim was to investigate long-term exposure to air pollution, the data allowed assessing exposure only one year back in time for the majority of our study subjects. However, the geographical contrasts in exposure over time are fairly constant. We therefore consider the annual mean of NO_x _the year before the stroke-date as a good proxy for NO_x _exposure over a longer time period.

A large proportion (78%) of the second-phase controls had not changed residential address during the past 10 years according to their answers in the public health survey. For the first-phase controls, we had no information on residential address back in time. Register data on residential address back in time was available for roughly half of the cases (based on what part of Scania they lived in). Although not entirely exact, that data indicated that the proportion of cases that had not changed residential address 10 years previous to the stroke was similar to the proportion of the second-phase controls (78%). Out of those we had information on, around 5% had changed residential address the year previous to the stroke year.

### Statistical analysis

The study was designed as a two-phase study, a design that can increase precision and reduce bias from selective participation in case-control studies.[27,28] In this study, the two-phase design was used to investigate whether residual confounding of the variables smoking, diabetes, and medication for hypertension occurred. These variables were only known for a subsample of the study subjects (the second-phase subjects), for 4 375 of the ischemic stroke cases and 4 716 of the controls (Table 2, Figure 2).

In a two-phase design, the log-odds ratio log(*ÔR*^1+2^) for the combined first- and second-phase data (1+2) can be estimated with adjustments for confounders in the following way.[27]

(1)

Logistic regression analysis including the variable birth year category, sex, marital status, and country of birth as covariates yielded ORs that were denoted as the "partly adjusted ORs". This corresponds to *ÔR*^1;*unadj *^and *ÔR*^2;*unadj *^in formula 1 for the first- and second-phase subjects, respectively, hereafter denoted *ÔR*^1;*Party_adj *^and *ÔR*^2;*Party_adj*^. The second-phase OR, adjusted also for smoking, diabetes, and medication for hypertension was denoted *ÔR*^2;*adj *^in formula 1 or as the "fully adjusted OR".

The NO_x _variable was analyzed both as a continuous measure and categorized as: 0–<10, 10–<20, 20–<30 and >30 μg/m^3^, with 0–<10 μg/m^3 ^as reference category. The p-value for the effect of the continuous NO_x _variable was used as a p-value for trend. The variance expression in formula 1 has been derived only for categorical variables; therefore we refrained from calculating the two-phase confidence intervals for the continuous NO_x _variable.

For the partly adjusted first-phase analysis, we explored potential effect modifications on the association between the continuous NO_x_-variable and ischemic stroke for the variables birth year category (> 1940 or ≤ 1940), sex, birth country and residing in urban/rural area. For the fully adjusted second-phase analysis, we explored effect modification by smoking status.

In order to take account for the different registration coverage between the hospitals, we applied a sampling technique for the controls in which they were assumed to be recruited with a probability according to the registration coverage in their hospital admission area. The data were analyzed as described by Weinberg and Wacholder,[29] (page 967–969), where an offset is added to the model to account for the "biased" sampling. The offset is calculated for each individual according to the probability of recruitment.

All analyses were done using SAS version 9.1 (SAS Institute Inc., Cary, NC).

## Results

The first-phase data and the ORs for the association between ischemic stroke and birth year, sex, marital status and birth country are presented in Table 1. The second-phase data and the ORs for the association between ischemic stroke and smoking, diabetes and medication for hypertension are presented in Table 2

We observed no associations between annual mean of NO_x _and risk of ischemic stroke, a result that was consistent between the partly adjusted first-phase analysis and the two-phase analysis (Table 3). The partly adjusted second-phase analysis, where selective participation is present among the controls, indicated an association (Table 3).

**Table 3:** Odds ratios (ORs) and confidence intervals (CIs) for ischemic stroke risk in relation to residential modelled outdoor annual mean concentration of NO_X_.

	First-phase analysis				Second-phase analysis						Two-phase analysis	
	
			Partly adjusted^a^				Partly adjusted^b^		Fully adjusted^c^			
								
NO_X_ μg/m^3^	Cases N(%)	Controls N(%)	OR^d^	CI	Cases N(%)	Controls N(%)	OR^e^	CI	OR^f^	CI	OR^g^	CI^h^
0–<10	1667 (34)	197371 (35)	1		1492 (34)	1784 (38)	1		1		1	
10–<20	1944 (40)	221981 (40)	1.03	0.97–1.10	1699 (39)	1923 (41)	1.07	0.91–1.25	1.00	0.85–1.2	0.97	0.90–1.05
20–<30	1065 (22)	110001 (20)	1.05	0.97–1.14	981 (22)	867 (18)	1.10	0.87–1.39	1.00	0.79–1.3	0.95	0.86–1.06
30–<60^i^	228 (5)	27559 (5)	0.91	0.79–1.05	203 (5)	142 (3)	1.28	0.90–1.82	1.20	0.85–1.76	0.87	0.73–1.03

^Partly adjusted first- and second-phase estimates, fully adjusted second-phase estimates and two-phase estimates: The two-phase estimates were calculated according to formula 1.^

^a ^Adjusted for birth year category, sex, birth country and marital status.

^b ^Adjusted for ^a ^and for geographical area.

^c ^Adjusted for ^a^, ^b ^and for smoking, diabetes and medication for hypertension.

^d ^The partly adjusted first-phase OR for an increase of 10 μg/m^3 ^in NO_x _was OR^1;*Partl_adj *^= 0.99 (0.95–1.02), p for trend = 0.44.

^e ^The partly adjusted second-phase OR for an increase of 10 μg/m^3 ^in NO_x _was OR^2;*Partly_adj *^= 1.09 (0.95–1.26), p for trend p = 0.21.

^f ^The fully adjusted second-phase OR for an increase of 10 μg/m^3 ^in NO_x _was OR^2;*Fully_adjj *^= 1.07 (0.93–1.24), p = 0.33

^g ^The two-phase OR for an increase of 10 μg/m^3 ^in NO_x _was OR^1+2 ^= 0.97.

^h ^The widths of the two-phase CIs are likely slightly overestimated, due to dependence between the three combined estimates that we did not take into account.

^i^Median level of NO_x _in the category 30–<60 μg/m^3 ^is 33.0 μg/m^3 ^(10^th^–90^th ^percentile: 30.4–40.5)

We observed no clear effect modifications with respect to age or sex. The partly adjusted first-phase *ÔR*^1;*Party_adj *^for a 10 μg/m^3 ^increase in NO_x _in individuals born 1923 through 1940 was 0.96 (0.92–1.00), and in persons born 1941 through 1965 it was 1.03 (0.95–1.10); p-value for no effect modification (testing the null hypothesis of no multiplicative interaction) = 0.13. The partly adjusted first-phase *ÔR*^1;*Party_adj *^for a 10 μg/m^3 ^increase in NO_x _in men was 0.96 (0.92–1.01), and in women it was 1.02 (0.96–1.07); p-value for no effect modification = 0.14.

The partly adjusted first-phase *ÔR*^1;*Party_adj *^for a 10 μg/m^3 ^increase in NO_x _was in urban areas 0.97 (0.89–1.07), and in rural areas 0.88 (0.82–0.94); p-value for no effect modification = 0.09.

The second-phase *ÔR*^2;*adj *^for the association between a 10 μg/m^3 ^increase in NO_x _and ischemic stroke was in smokers 0.92 (0.76–1.11) and in non-smokers 1.11 (0.95–1.28): p-value for no effect modification p = 0.01. Converting the two-phase *ÔR*^1+2 ^of 0.97 to account for effect modification by smoking (assuming a population of 20% smokers and 80% non-smokers) yields an *ÔR*^1+2 ^point estimate of 0.92 for smokers and of 1.00 for non-smokers.

In a sub-analysis, we limited our analysis to only include cases with a 30-day or less survival after the stroke (N = 244), yielding *ÔR*^1;*Party_adj *^of 0.96 (0.82–1.13) for a 10 μg/m^3 ^increase in NO_x_.

Effect modification by birth country was not evident in this study, p = 0.89. The *ÔR*^1;*Party_adj *^associated with a 10 μg/m^3 ^increase in NO_x _was 0.99 (0.95–1.03) for persons born in Sweden, *ÔR*^1;*Party_adj *^= 1.02 (0.87–1.19) for persons born in the other Nordic countries and *ÔR*^1;*Party_adj *^= 0.97 (0.87–1.08) for persons born in the rest of the world.

## Discussion

### Main results

Hospital admissions for ischemic stroke in Scania were not associated with long-term outdoor NO_x _at the residential address. No clear effect modification with respect to age or sex was observed.

### Strengths and limitations of the study

The main strengths of the present study are the large and almost complete number of cases (N = 4 904), the complete study base, and the high-quality exposure assessment of residential outdoor NO_x_.

Even so, this study had limitations. As in all epidemiologic studies of this kind, and although our exposure assessment was of high quality, there was exposure measurement error leading to exposure misclassification.

Zeger and colleagues discuss three components of exposure measurement error in time-series on air pollution data:.[30] 1) Error due to aggregation of exposure, 2) Difference between average personal exposure and true ambient level, and 3) Difference between true and measured ambient level. Since we study long-term rather than short-term exposure, we reformulated "3", to be the difference between modelled and true ambient NO_x_-concentrations and added another component of the error, 4) Error caused by migration.

1) Aggregated (500 × 500 spatial resolution) rather than individual exposure estimates leads to a Berkson type error that should not lead to bias in linear models and generally only marginal bias in log-linear models.[30]

2) Concentrations outside a residence (where we model NO_x_) do not necessarily correlate well with levels inside, or with actual exposure.[31] We did not have data on time spent at home or on other sources of exposure, except for data on smoking status for the second-phase subjects. Although smoking is a major source of indoor air pollution, adjusting for it did not alter the effect estimates substantially (Table 3). Other differences between between actual and ambient exposure most likely cause bias towards the null, but it is hard to estimate the magnitude. By adjusting adequately for socio-economic factors, which has a complex relationship to air pollution,[18] and likely a large influence on other potential exposures to air pollution, bias caused by this component of measurement error might be reduced.

3) The validation between modelled and measured levels indicated a measurement error of classical type, typically also yielding bias towards the null. If we assume that the true effect is OR = 1.10 for a 10 μg/m^3 ^increase in NO_x_, and assume that the measurements accurately reflect the true levels, we can apply the formula by Armstrong[32,33] to calculate the expected OR as  = 1.09, where the ratio in the exponent is given by the measured ("true") variance, V_T _= 7.7^2 ^and the modelled (observed) variance, V_obs _= 8.3^2^. Thus, for modest effects, the bias caused by this error component does not seem to be substantial

4) The proportion that had not migrated (estimated to 78%) within 10 years before the stroke year was similar between cases and second-phase controls, again implying bias towards the null.

If we conservatively assume that the error in the 22% that migrated is completely of classical type and the variance in those 22% is double to the true variance (*V*_*T*_) in the 78% that did not migrate. For a true effect of OR = 1.10 per 10 μg/m^3 ^increase in NO_x_, we get the expression: , a moderate bias.

In summary, the error components for which we could estimate the impact did not seem to substantially influence the effect estimates. However, we cannot rule out that differences between actual personal exposure and ambient exposure may have yielded more substantial bias. Also, exposure measurement error would yield more substantial bias if the true OR was larger than 1,1, say for example 1.3. Point 4 above would then cause bias to an OR of 1.23 under our conservative assumption.

Another source of a (slight) dilution of the effect estimates was the fact that we were unable to link the national stroke register to the Scanian population, which means that a small fraction of the controls may have been cases.

The correlation between modelled NO_x _and experienced disturbance from air pollution was high in the public health survey, which strengthens the validity of our residential exposure assessments.[34]The proportion that was disturbed by air pollution near their residence was 53% in the highest NO_x_-category (≥30 μg/m^3^), 36% in the NO_x_-category 20–<30 μg/m^3^, 18% in the NO_x_-category 10–<20 μg/m^3 ^and 14% in the lowest NO_x_-category (<10 μg/m^3^).

The biased sampling analysis, where differences in coverage between the hospital admission areas were accounted for, indicated that difference in coverage between hospitals did not substantially influence the effect estimates in this study, an *ÔR*^1;*Party_adj *^of 0.98 (0.94–1.02).

For the two-phase analysis, the participants in the public health survey of 2004 provided a sample of potential controls with an already available data set on important confounding factors. There was selective participation in the public health survey with respect to income, age, education, sex, marital status and country of birth. There was also a group-level negative correlation between degree of participation and air pollution, which suggests a bias away from the null. The second-phase analysis suggests an overestimation of the effect (although not statistically significant): the partly adjusted *ÔR*^2;*unadj *^for a 10 μg/m^3 ^increase in NO_x _was 1.09 (0.95–1.26) whereas the partly adjusted first-phase *ÔR*^1;*Party_adj *^was 0.99 (0.95–1.02). We note that using only second-phase data would in this study perhaps have led to false conclusions due to the selective participation of the second-phase controls and that the two-phase design is a strength of this study.

The indication of a protective effect by NO_x _in rural areas, OR = 0.88 (0.82–0.94) is hard to explain apart from it might be a chance finding. We do not see evidence in our data for residual confounding or biased exposure estimates being plausible explanations for this seemingly protective effect. When adjusting for three of the most important risk factors for stroke (diabetes, smoking, hypertension), the effect estimates change only slightly (Table 3). We find it unlikely that strong residual confounding remains only in the rural areas. In addition, it is also unlikely that exposure measurement errors would cause bias below null, since reversal of the direction requires both that the variance of the error is larger than the variance of the true error and a strong negative correlation between the error and the true value.[25]

### The results in relation to previous studies

Long-term effects on stroke risk have been observed previously in areas where levels of air pollution were considerably higher than in this study. In the UK, an increase in stroke-related mortality of 37% was observed between the lowest and highest quintile group of modelled NO_x _(mean 47.6 μg/m^3 ^and 61.9 μg/m^3^, respectively).[10] Miller and colleagues from USA reported a hazard ratio for first-ever cerebrovascular events (where cerebrovascular deaths were included) in women of 1.35 associated with a 10 μg/m^3 ^increase in PM_2.5_, where the mean value of PM_2.5 _was 13.5 μg/m^3^.[11] This corresponds to higher levels of air pollution than in our study area. The hazard ratio for cerebrovascular deaths, which consisted of 20% of the cerebrovascular events, was 1.83 (1.11–3.00). Miller and colleagues recorded first-ever events, as we do in this study, and used monitoring sites to asses exposure. In the Norwegian study, which found an OR for cerebrovascular mortality of 1.04 (0.94–1.15) associated with a 10 μg/m^3 ^increase in modelled NO_x_, the levels of NO_x _were rather similar to the levels in Scania, although the composition of air pollution has most likely changed substantially between the 1970s (Norwegian study) and the 2000s (our study).

Perhaps other factors, similar in the Norwegian and Swedish populations, account for the similar results of the two studies. One of those factors might be the rather low concentrations of air pollution, perhaps yielding a larger influence from potential exposure measurement error, though in the Norwegian study, other outcomes showed clear associations with air pollution concentrations.[12]

A Swedish study on myocardial infarction and long-term exposure to air pollution indicated an effect on out-of-hospital deaths but not on non-fatal cases.[35] We could not include out-of-hospital deaths in this study, and we restricted our cases to first-ever strokes. The proportion of out-of-hospital deaths seems small in our population (1% in one of the hospital admission areas), the group of male stroke patients with mild strokes who were sent home without being admitted to the hospital was larger (8%). We cannot rule out a potential effect in those groups of stroke cases, or in the rather small fraction of stroke cases that for some reason were not registered in the regional stroke register. However, we see no tendency for a more pronounced effect in cases with a short survival after stroke (30 days or less), with a first-phase OR of 0.96 (0.82–1.13) associated with a 10 μg/m^3 ^increase in NO_x_.

The mean age at diagnosis of the study subjects in this study was rather low (69 years). We decided to include only individuals who were born between 1923 and 1965, since in an older population, competing risks are a problem when making inferences about exposure. Also, the second-phase control material was restricted to individuals born 1923 and later and thus, we did not have the opportunity to adjust for important risk factors in a population born before 1923. Without any age- or first-time stroke restrictions, the mean age in our case group was 78 years (range 3–103 years). Studies on air pollution and stroke often lack an upper age restriction, especially for short-term exposure,[3-6,8] but also for long-term exposure.[9,10] The associations observed in many studies on short-term exposure to air pollution may be partly attributable to the (older) population studied. The Norwegian study (which did not observe an association between long-term exposure to air pollution and cerebrovascular mortality) followed males aged 42–75 years.[12] The USA (positive) study included women aged 50–79 years.[11] In our study, we observe no statistically significant modification of effect by sex or age.

Thus, there seem to be evidence for an effect by long-term exposure to air pollution on cerebrovascular mortality, where air pollution concentrations are higher than in our study area. To our knowledge, this study is the first to explore hospital admissions for ischemic stroke in association with long-term exposure to air pollution. The only other study on long-term exposure to air pollution and cerebrovascular events also includes cerebrovascular mortality.[11] In that study the hazard ratio decreased in the mixed group (cerebrovascular events and cerebrovascular deaths: Hazard ratio = 1.35) compared to the group with only cerebrovascular deaths (Hazard ratio = 1.83). The evidence is therefore less clear for cerebrovascular hospital admissions than for cerebrovascular mortality.

### Generalizability

How applicable are our estimates to other populations? Air pollution is not a static concept, but it differs in composition between regions. Comparison of risk estimates between regions can therefore be complicated. Generalizability is especially problematic as individual exposure to air pollution is assessed with a proxy for air pollution, in this study, modelled levels of NO_x_. The results in this study should rather be seen as an indicator of the risk caused mainly by traffic-related air pollution in rather low-level areas, although misclassification of exposure might have yielded falsely negative results. The study population was all individuals born between 1923 and 1965 in the entire region of Scania, a complete study base, which makes the generalizability of the study high. Given the large and complete study population and case series, the results could probably be extrapolated to other similar populations, to describe the effects of traffic-related air pollution at low levels.

### Issues for future research

Using the second-phase data, we observed an effect modification by smoking status, which suggested an effect by an increase of 10 μg/m^3 ^in NO_x_on ischemic stroke risk for non-smokers but not for smokers. Converted to the two-phase estimate, the increase in risk for non-smokers was no longer present (OR^1+2 ^= 1.00). However, the idea that smokers would be less sensitive to air pollution than non-smokers is interesting, and should be further explored.

In a negative study, it is of great importance to rule out potential sources of error that might lead to bias towards the null. We concluded that although our exposure assessment was of very high quality, exposure misclassification might have biased our results towards the null. Adjusting adequately for socio-economic factors might reduce that bias. Researchers in low-level areas aiming at establishing whether an association between long-term exposure to air pollution and ischemic stroke risk have a challenge in reducing misclassification of exposure.

## Conclusion

In this large study, in an area where levels of air pollution are generally rather low, with a complete study base, high-quality case ascertainment, and individually modelled exposure, we observed no evidence of an association between long-term exposure to NO_x _(as a marker for air pollution) and hospital admissions for ischemic stroke.

## Competing interests

The authors declare that they have no competing interests.

## Authors' contributions

AO participated in the design of the study, performed the statistical analysis and drafted the manuscript. ES carried out the GIS analysis and helped to draft the manuscript. US, KJ and JB conceived of the study, and participated in its design and coordination and helped to draft the manuscript. All authors read and approved the final manuscript.

## Funding

This study was supported with grants from the Swedish emission research programme (EMFO) and from Lund University.

## Pre-publication history

The pre-publication history for this paper can be accessed here:


